# Alendronate treatment rescues the effects of compressive loading of TMJ in osteogenesis imperfecta mice

**DOI:** 10.1186/s40510-024-00526-2

**Published:** 2024-07-15

**Authors:** Po-Jung Chen, Shivam Mehta, Eliane H. Dutra, Sumit Yadav

**Affiliations:** 1 Department of Growth and Development, College of Dentistry, University of Nebraska Medical Center, Lincoln/Omaha, NE USA; 2 Department of Orthodontics, School of Dentistry, Texas A&M University, Dallas, TX USA; 3 Division of Orthodontics, School of Dental Medicine, UConn Health, Farmington, CT USA; 4 Department of Growth and Development, College of Dentistry, University of Nebraska Medical Center, Lincoln/Omaha, NE USA

**Keywords:** Osteogenesis imperfecta, Temporomandibular joint, Alendronate, Mandibular condylar cartilage

## Abstract

**Background:**

Osteogenesis imperfecta (OI) is a genetic disorder of connective tissue caused by mutations associated with type I collagen, which results in defective extracellular matrix in temporomandibular joint (TMJ) cartilage and subchondral bone. TMJ is a fibrocartilaginous joint expressing type I collagen both in the cartilage and the subchondral bone. In the present study the effects of alendronate and altered loading of the TMJ was analyzed both in male and female OI mice.

**Materials and methods:**

Forty-eight, 10-weeks-old male and female OI mice were divided into 3 groups: (1) Control group: unloaded group, (2) Saline + Loaded: Saline was injected for 2 weeks and then TMJ of mice was loaded for 5 days, (3) alendronate + loaded: alendronate was injected for 2 weeks and then TMJ of mice was loaded for 5 days. Mice in all the groups were euthanized 24-h after the final loading.

**Results:**

Alendronate pretreatment led to significant increase in bone volume and tissue density. Histomorphometrically, alendronate treatment led to increase in mineralization, cartilage thickness and proteoglycan distribution. Increased mineralization paralleled decreased osteoclastic activity. Our immunohistochemistry revealed decreased expression of matrix metallopeptidase 13 and ADAM metallopeptidase with thrombospondin type 1 motif 5.

**Conclusion:**

The findings of this research support that alendronate prevented the detrimental effects of loading on the extracellular matrix of the TMJ cartilage and subchondral bone.

## Introduction

Osteogenesis imperfecta (OI), also known as brittle bone disease, is a genetic disease of connective tissue that is characterized by low bone mass, and varying degrees of bone fragility which predisposes the patient to spontaneous fracture [1–5]. It is a life-long disorder, distributed equally among males and females and in all ethnic groups [5]. More than 90% of OI cases are autosomal dominant, caused by mutations in the COL1A1 and COL1A2 genes which code for the α1(I) and α2(I) chains of Type I collagen, altering the protein structure or quantity [2–5].

There is no cure for OI currently and bisphosphonates are the drug commonly prescribed to the individuals with OI [6]. Bisphosphonates are potent anti-resorptive agent that inhibits the functions of osteoclast [6]. Pamidronate and alendronate (ALN) have been shown to be effective in treating different subtypes of OI, as they significantly increase bone mineral density and decrease bone turnover [7–10].

The temporomandibular joint (TMJ) is a unique diarthrodial, synovial joint formed by the articulation of the mandibular condylar cartilage (MCC), the TMJ disc, and the cartilage of the glenoid fossa [11,12]. The MCC, the TMJ disc and the cartilage of the glenoid fossa are fibrocartilaginous and predominantly consist of chondrocytes which expresses type 1 collagen [11–13]. Furthermore, osteoblasts in the subchondral bone of the condyle expresses type 1 collagen [11,12].

Osteogenesis imperfecta murine (*oim*) mice, one of the most commonly used transgenic mouse models for OI, are a phenocopy of the naturally occurring mutation causing human type III OI [14]. The mutation in *oim* mice is a single nucleotide deletion (G) that alters the terminal approximately 50 amino acids of the pro-alpha 2 C-propeptide and prevents association with the pro-alpha 1 chains. As a result, α1(I) collagen homotrimers, instead of normal heterotrimer α1(I)2α1(I) collagen, could be produced in *oim* mice [15,16]. The *oim* model has been widely used to examine the mechanical properties of long bones at the tissue and cellular level and to develop therapeutics [17]. Surprisingly, no research has focused on studying the MCC and the subchondral bone in the *oim*, though both the tissues predominantly express type 1 collagen.

Therefore, the goal of this study was to characterize the osteochondral tissue (mandibular condylar cartilage and the subchondral bone) of TMJ in OI mice, and to determine the effects of bisphosphonates (alendronate) on the osteochondral tissue of TMJ during loading. In addition, since both male and female *oim* mice were analyzed, we also aim to investigate whether the TMJ phenotype and effects of alendronate on loading are gender-dependent.

## Materials and methods

### Transgenic mice

All the experimental procedures were approved by the Institutional Animal Care and Use Committee (IACUC) at University of Connecticut Health Center. Homozygous *oim* mice (*oim/oim*, Jackson laboratory, stock # 001815) of both genders were used to study the histological and mechanical characteristics of the osteochondral tissues of the TMJ, as well as the effects of bisphosphonates on these osteochondral tissues under compressive loading.

### Study design

**Fig. 1:**
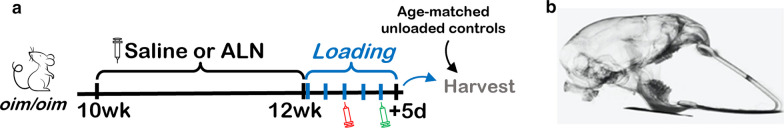
**a** Schematic of experiments. **b** Compressive loading model of TMJ

The 10-weeks-old male and female homozygous *oim* mice were randomly allocated to: (1) Control Group (8 male + 8 female); (2) Saline + Loaded (8 male + 8 female): mice were injected with saline every alternate day for 2 weeks, immediately followed by 5 days of functional loading. (3) ALN + Loaded (8 male + 8 female): mice were injected with ALN (alendronate sodium trihydrate, 50 μg/kg, Sigma Aldrich, St. Louis, MO) every alternate day for 2-weeks, immediately followed by 5 days of functional loading (Fig. 1a). The mice in groups (2) and (3) were loaded for one hour each day for 5 days (Fig. 1b). Twenty-four hours after the last loading, all the loaded and control mice were sacrificed.

### Functional loading model and fluorochrome labeling

The osteochondral tissue of the *oim* male and female mice in the Saline + Loaded and ALN + Loaded group were loaded in a compressive fashion using an established protocol [18–22]. Briefly, the *oim* mice were anesthetized with a mixture of ketamine (90 mg/kg) and Xylazine (13 mg/kg), then loaded with the customized spring made from Connecticut New Archform (CNA) wire (0.32inch), which delivered a force of 0.5N as measured by the Correx tension gauge (Haag-Streit, Bern, Switzerland).

All the mice in the control and experimental groups were injected intraperitoneally with alizarin red (Sigma, A3882, 20 mg/kg body weight) on the third day, and calcein (Fluka, 21,030, 10 mg/kg body weight) on the fifth day 3 h prior to sacrifice.

### Micro-computed tomography (micro-CT) analysis

Mandible samples (n = 8 per group) were scanned with a μCT40 instrument (Scanco Medical AG, Bruttisellen, Switzerland). The technician performing scans and analysis was blinded to the treatment groups. Serial tomographic projections were acquired at 55 kV and 145μA, with a voxel size of 6 μm and 1000 projections per rotation were collected at 300,000 μs. The region of interest was the mushroom-shaped condylar head which includes the MCC and the subchondral bone. Bone volume fraction (BVF, %) and tissue density (TD, mg/ccmHA) were analyzed.

### Tissue preparation, histology, and immunostaining

The condyles were dissected and fixed in 4% formaldehyde for 48 h, then transferred to 30% sucrose overnight and embedded in cryomedium (Thermo Shandon, Pittsburgh, PA, USA). Sagittal sections of the MCC along with the subchondral bone (5 μm-thick) were cut using a Leica cryostat (Nussloch, Germany). Our histological sections were stained and analyzed as previously described [23]. The sections were first imaged for the bone labels including alizarin red (red) and calcein (green). Baseline imaging of the sections was performed with an observer ZI fluorescent microscope (Carl Zeiss, Thornwood, NY, USA) using a cyan fluorescent protein filter (CFP, Chroma Cat 49001ET, EX: 436/20, EM: 480/40) and a RFPcherry filter that was also used for detecting alizarin red labeling (mCherry, Chroma Cat 49009ET, EX: 560/40, EM: 630/75). Sections were then stained for Tartrate Resistant Acid Phosphatase (TRAP) using ELF97 (Life Tech, Waltham, MA, USA), generating a yellow fluorescent signal. Afterwards, the coverslip was removed and stained for cell nuclei using DAPI (Thermo Fisher Scientific, Waltham, MA, USA) then reimaged. Finally, the slide was rinsed in distilled water then stained with Toluidine Blue (TB) and reimaged using bright field microscopy to examine the proteoglycans.

We also performed Safranin O staining, as well as immunohistochemistry for degeneration markers, including matrix metallopeptidase 13 (MMP13; Abcam, Cambridge, MA, USA) and ADAM metallopeptidase with thrombospondin type 1 motif 5 (ADAMTS5; Thermo Fisher Scientific, Waltham, MA).

### Histomorphometric analyses

ImageJ (National Institutes of Health, Bethesda, MD) was used to quantify all the histological staining used in this research. We examined TRAP activity in MCC and subchondral bone by counting the number of yellow pixels and dividing by the total number of pixels in the subchondral bone region. Cartilage thickness was analyzed on the Toluidine Blue stained sections and was measured at three different points from anterior to posterior. Similarly, proteoglycan distance was quantified on the Safranin O sections and was measured at eight different regions from anterior to posterior. MMP13 and ADAMTS5 was quantified by percentage of red positive cells over DAPI positive cells.

### Statistical analyses

All the data are expressed as mean ± SD. Differences among the control and two loaded groups of both genders were determined by two-way analysis of variance (ANOVA) with Tukey’s post-hoc test. All statistical tests were two sided and a *p*-value of < 0.05 was deemed to be statistically significant. Statistical analyses were computed using Graph Pad Prism (San Diego, CA, USA).

## Results

### Bone volume and remodeling

**Fig. 2:**
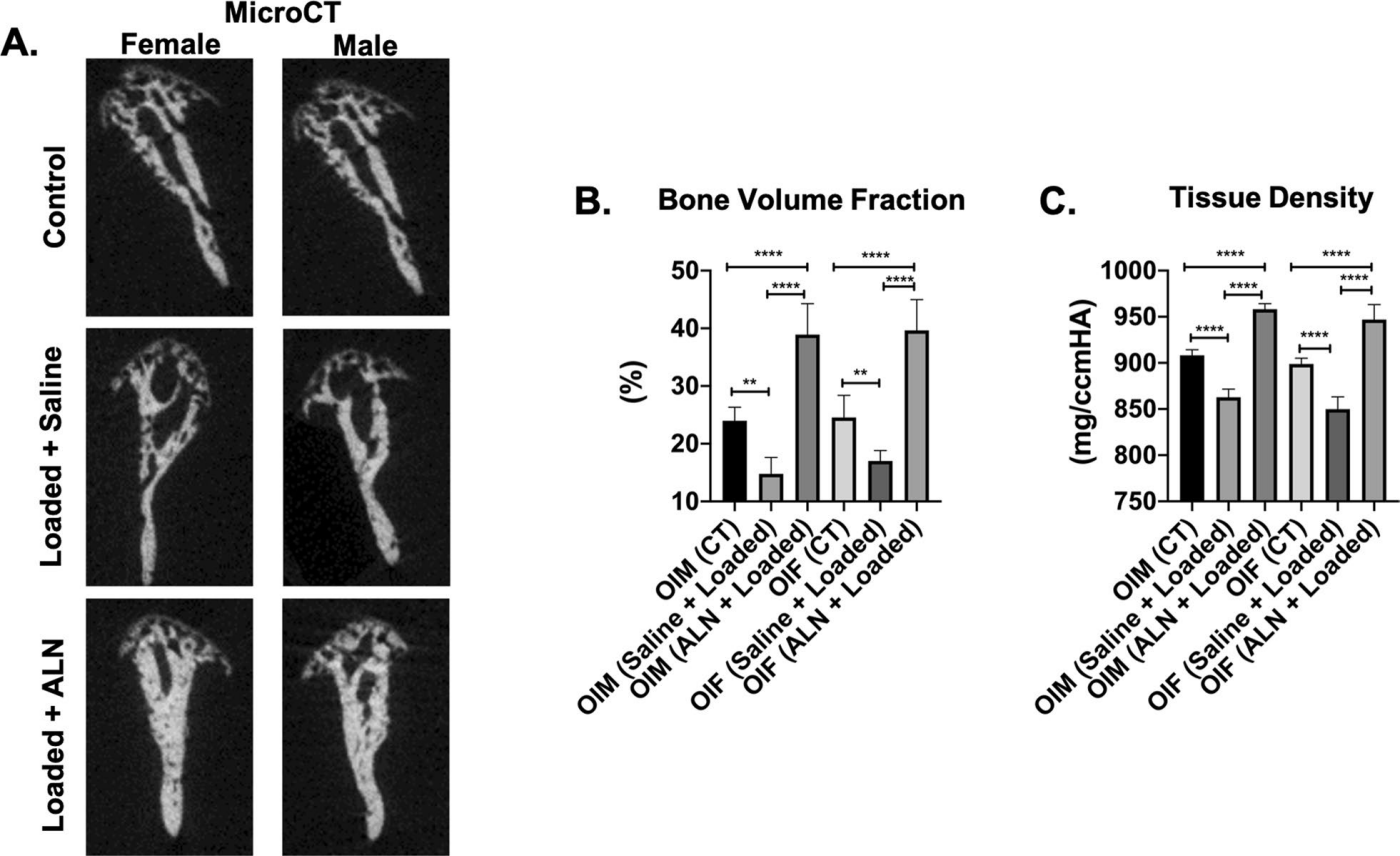
Significant increased bone volume with mechanical loading and alendronate (ALN) treatment in OI mice. **a** Coronal micro–computed tomography (micro-CT) images of condyles of control and experimental groups (Saline + Loaded and ALN + Loaded), **b** bone volume fraction, **c** tissue density. Histograms (**b, c**) represent means ± standard deviation (SD) for *n* = 8 per group. Statistically significant difference between groups: **P < 0.001, ****P < 0.0001. OIM: Osteogenesis imperfecta male; OIF: Osteogenesis imperfecta female

Upon altered loading, the bone volume fraction (BVF) and tissue density (TD) of the TMJ subchondral bone were significantly decreased in both genders when compared to the unloaded control (BVF: Saline + Loaded vs Control: *p* < 0.0001; Saline + Loaded vs ALN + Loaded: *p* < 0.0001; Fig. 2a, b. TD: Saline + Loaded vs Control: *p* < 0.0001; Saline + Loaded vs ALN + Loaded: *p* < 0.0001; Fig. 2a, c), indicating a catabolic effect of altered loading to the subchondral bone. However, when given alendronate prior to loading, this catabolic effect was prevented, as revealed by a significantly higher BVF and TD in ALN + Loaded group than in Saline + Loaded group (*p* < 0.0001); in fact, the alendronate pretreatment led to an even denser and stronger subchondral bone than the unloaded group (BVF and TD: *p* < 0.0001). Therefore, these data suggest a detrimental effect of altered loading on the TMJ subchondral bone volume and density, which could be prevented by the administration of alendronate prior to loading.

**Fig. 3:**
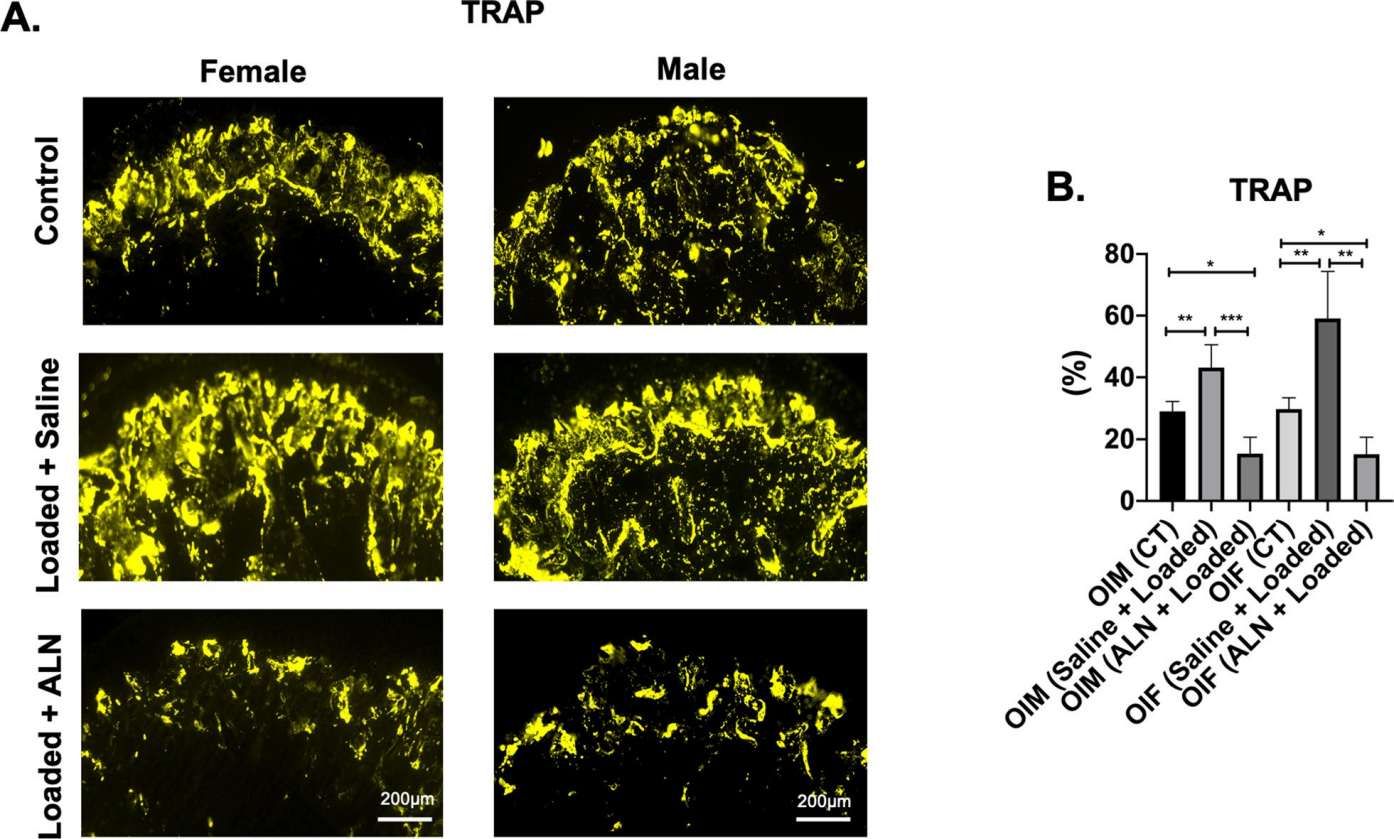
Significant decreased TRAP (Tartrate resistant acid phosphatase) with mechanical loading and alendronate (ALN) treatment in OI mice. **a** Sagittal sections of osteochondral tissue of TMJ stained for TRAP in control and experimental groups (Saline + Loaded and ALN + Loaded), **b** Quantification of TRAP. Histograms (**b**) represent means ± standard deviation (SD) for *n* = 6 per group. Statistically significant difference between groups: *P < 0.05, **P < 0.001, ***P = 0.0001. **a**. Scale bar = 200 µm. OIM: Osteogenesis imperfecta male; OIF: Osteogenesis imperfecta female

Similarly, altered loading of TMJ led to increased osteoclastic activity in Saline + Loaded group when compared to ALN + Loaded group and unloaded control group (*TRAP:* Saline + Loaded vs Control: *p* < 0.01; Saline + Loaded vs ALN + Loaded: *p* < 0.0001; Fig. 3a, b). *Oim* mice have inherent increased osteoclastic activity to remove the defective matrix and loading of TMJ accentuates the remodeling of the subchondral bone. In this light, our result signifies that ALN treatment before TMJ loading significantly reduces the osteoclastic activity which might have alleviated the bone loss observed in Saline + Loaded group (Fig. 3a, b**).**

**Fig. 4:**
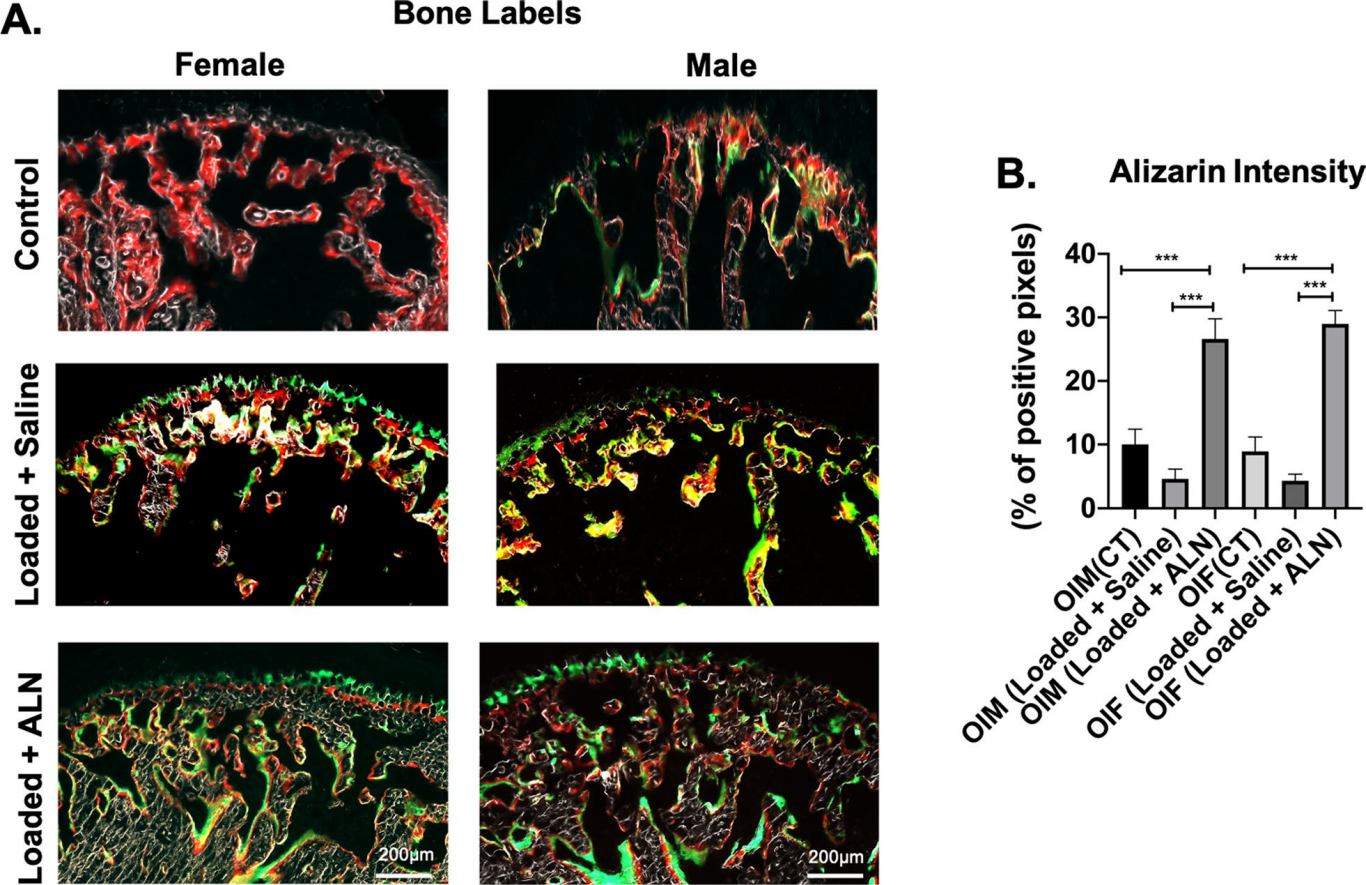
Significant increased alizarin red intensity (bone formation) with mechanical loading and alendronate (ALN) treatment in OI mice. **a** Sagittal sections of osteochondral tissue of TMJ stained for bone labels in control and experimental groups (Saline + Loaded and ALN + Loaded), **b** quantification of alizarin red intensity. Histograms (**b**) represent means ± standard deviation (SD) for *n* = 6 per group. Statistically significant difference between groups: ***P = 0.0001, ****P < 0.0001. **a**. Scale bar = 200 µm. OIM: Osteogenesis imperfecta male; OIF: Osteogenesis imperfecta female

Interestingly, our mineral apposition data parallels our BVF and osteoclastic activity data in both genders. Subsequent to altered loading of TMJ, mineral deposition as measured by alizarin intensity was significantly reduced in Saline + Loaded group when compared to ALN + Loaded group and unloaded control group (*Alizarin intensity*: Saline + Loaded vs Control: *p* < 0.01; Saline + Loaded vs ALN + Loaded: *p* < 0.0001; Fig. 4a, b). Notably, our data suggest that ALN not only have an effect on bone resorption/osteoclastic activity but also indirectly affects bone formation as revealed in significant increased alizarin intensity with pretreatment of ALN.

### Cartilage thickness and proteoglycan distance

**Fig. 5:**
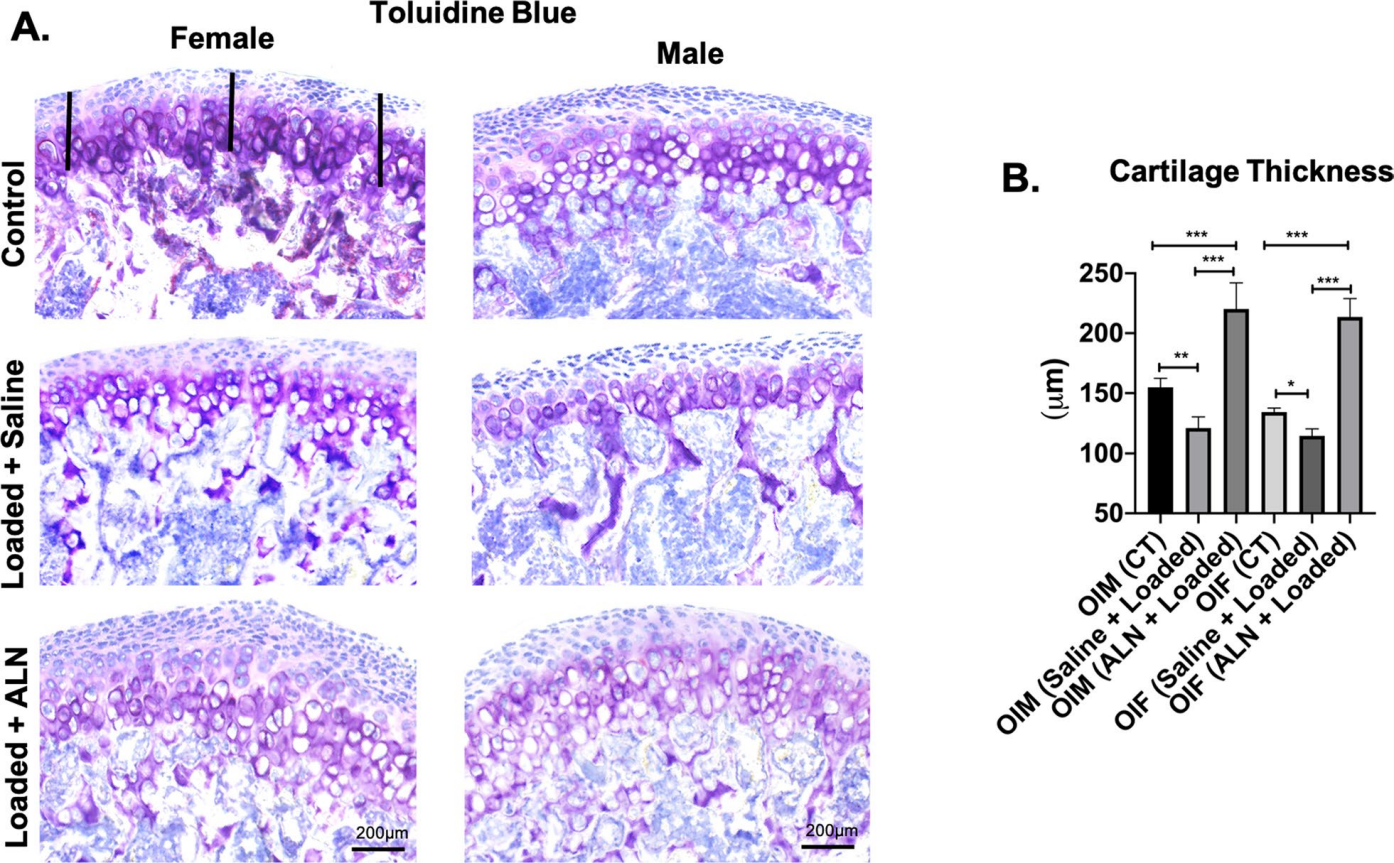
Significant increased cartilage thickness with mechanical loading and alendronate (ALN) treatment in OI mice. **a** Sagittal sections of osteochondral tissue of TMJ stained for Toluidine Blue in control and experimental groups (Saline + Loaded and ALN + Loaded), **b** Quantification of cartilage thickness. Histograms (**b**) represent means ± standard deviation (SD) for *n* = 6 per group. Statistically significant difference between groups: **P < 0.001. (**a**). Scale bar = 200 µm. OIM: Osteogenesis imperfecta male; OIF: Osteogenesis imperfecta female

**Fig. 6:**
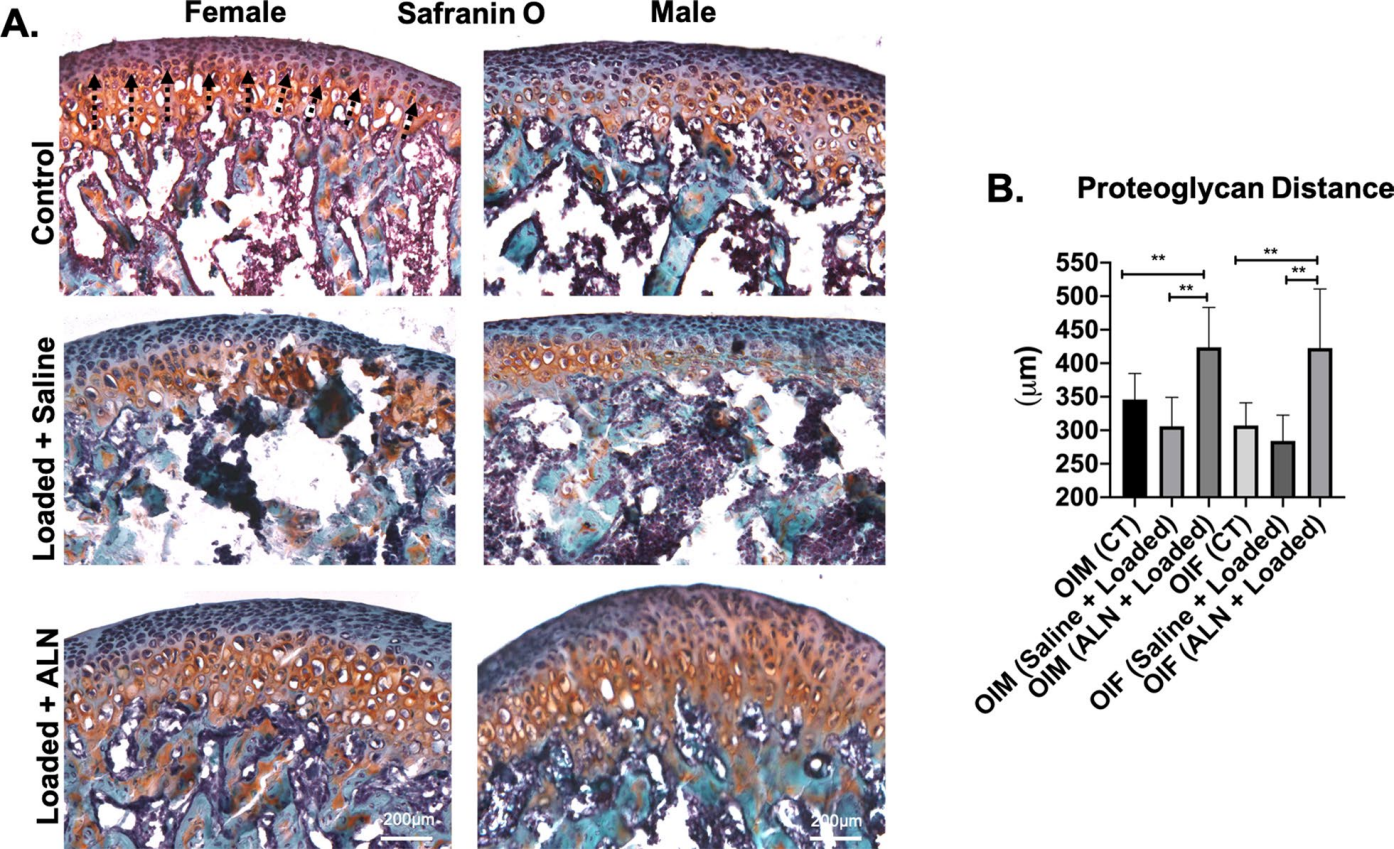
Significant increased proteoglycan distance (Safranin O staining) with mechanical loading and alendronate (ALN) treatment in OI mice. **a** Sagittal sections of osteochondral tissue of TMJ stained for Safranin O in control and experimental groups (Saline + Loaded and ALN + Loaded), **b** Quantification of proteoglycan distance from the tidemark. Histograms (**b**) represent means ± standard deviation (SD) for *n* = 6 per group. Statistically significant difference between groups: *P < 0.05, **P < 0.001, ***P = 0.0001. (**a**). Scale bar = 200 µm. OIM: Osteogenesis imperfecta male; OIF: Osteogenesis imperfecta female

The altered loading of the TMJ led to significantly decreased cartilage thickness and proteoglycan distance in Saline + Loaded group when compared to ALN + Loaded group and unloaded control in both genders (*cartilage thickness* Saline + Loaded vs Control: *p* < 0.05; Saline + Loaded vs ALN + Loaded: *p* < 0.0001; Fig. 5a, b. *proteoglycan distance* Saline + Loaded vs Control: *p* < 0.0001; Saline + Loaded vs ALN + Loaded: *p* < 0.0001; Fig. 6a, b), thus predisposing the cartilage to early breakdown. Nonetheless, ALN pretreatment prevented the cartilage to breakdown with altered loading of TMJ, as indicated by significant increased cartilage thickness and proteoglycan distance (*cartilage thickness*: *p* < 0.0001 and *proteoglycan distance:* *p* < 0.004). These data suggest that ALN treatment improves the load bearing capacity of the cartilage and prevents the predisposition to early breakdown.

### Expression of cartilage degeneration markers

**Fig. 7:**
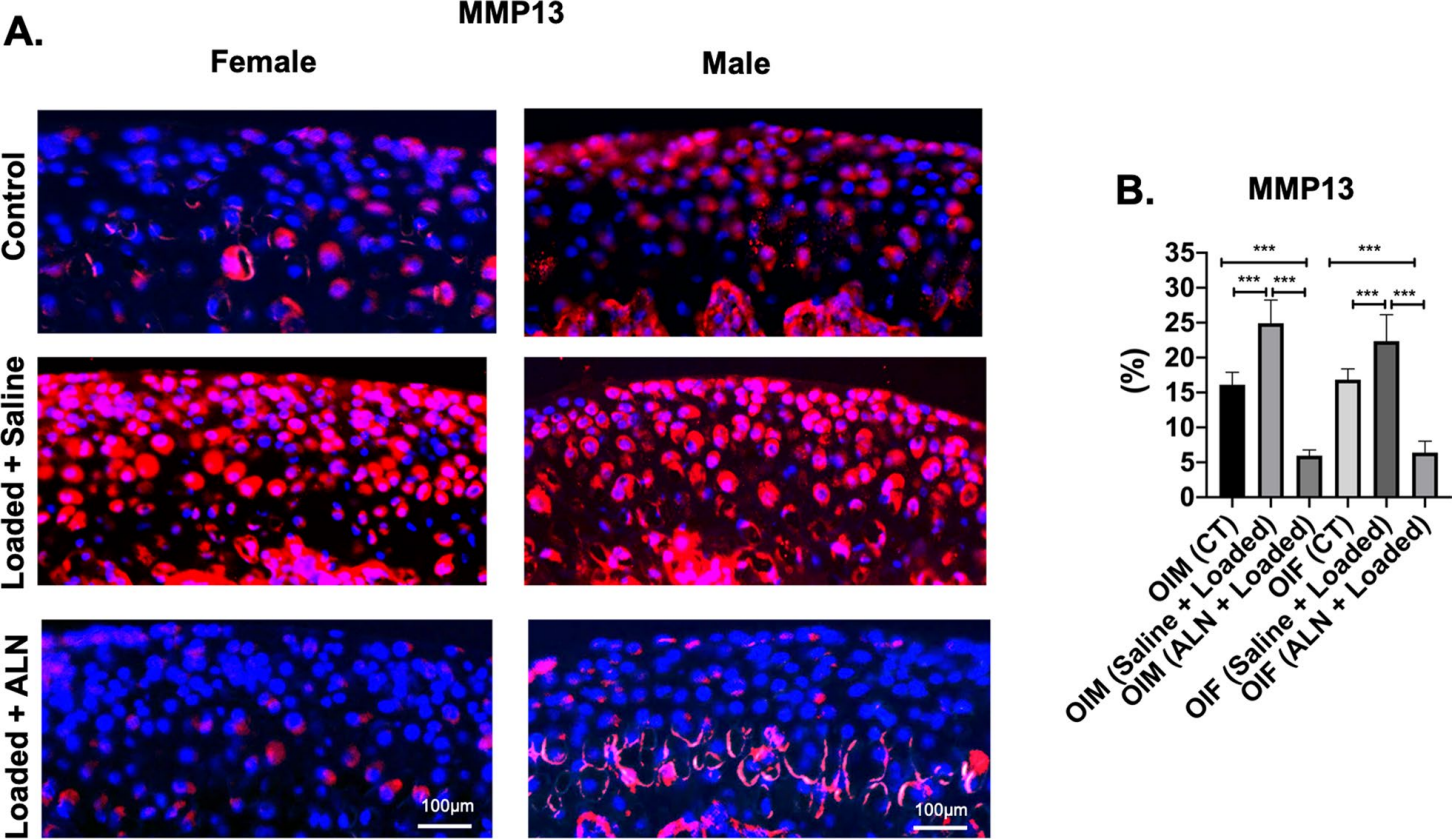
Significant decreased MMP13 expression with mechanical loading and alendronate (ALN) treatment in OI mice. **a** Sagittal sections of osteochondral tissue of TMJ stained for MMP13 in control and experimental groups (Saline + Loaded and ALN + Loaded), **b** Quantification of MMP13 (percentage of red positive cells over DAPI positive cells). Histograms (**b**) represent means ± standard deviation (SD) for *n* = 5 per group. Statistically significant difference between groups: *P < 0.05, **P < 0.001, ***P = 0.0001. (**a**). Scale bar = 100 µm. OIM: Osteogenesis imperfecta male; OIF: Osteogenesis imperfecta female

**Fig. 8:**
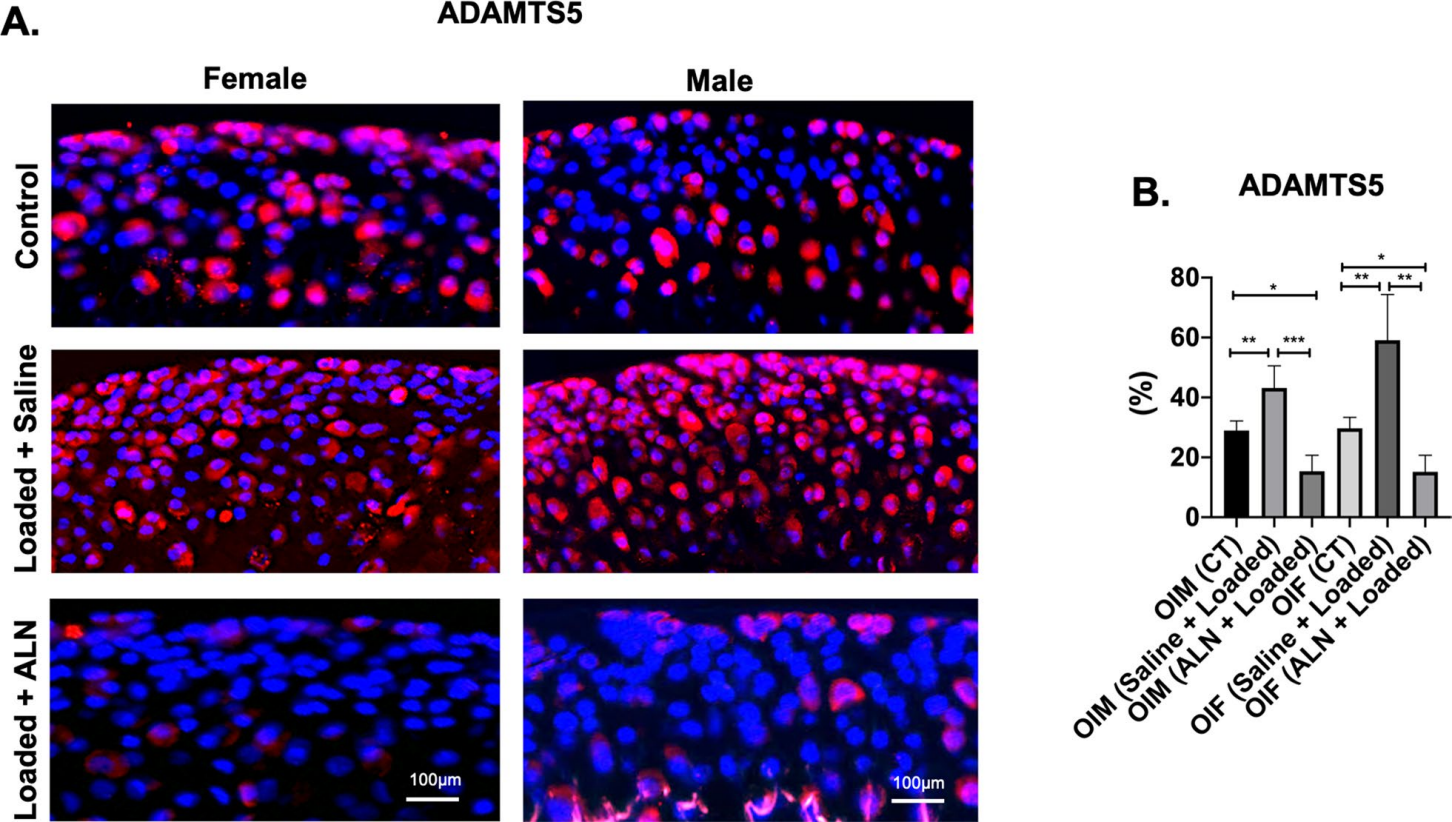
Significant decreased ADAMTS5 expression with mechanical loading and alendronate (ALN) treatment in OI mice. **a** Sagittal sections of osteochondral tissue of TMJ stained for ADAMTS5 in control and experimental groups (Saline + Loaded and ALN + Loaded), **b** Quantification of ADAMTS5 (percentage of red positive cells over DAPI positive cells). Histograms (**b**) represent means ± standard deviation (SD) for *n* = 5 per group. Statistically significant difference between groups: ***P = 0.0001, ****P < 0.0001. (**a**). Scale bar = 100 µm. OIM: Osteogenesis imperfecta male; OIF: Osteogenesis imperfecta female

Consequent to altered TMJ loading, MMP13 and ADAMTS5 were significantly increased in both genders when compared to the ALN + Loaded and unloaded control (*MMP13* Saline + Loaded vs Control: *p* < 0.05; Saline + Loaded vs ALN + Loaded: *p* < 0.0001; Fig. 7a, b. *ADAMTS5* Saline + Loaded vs Control: *p* < 0.0001; Saline + Loaded vs ALN + Loaded: *p* < 0.0001; Fig. 8a, b), indicating cartilage degeneration due to degradation of collagen matrix (MMP13) and depletion of proteoglycan and aggrecan (ADAMTS5). However, administration of ALN prior to loading, suppressed the effects of cartilage degeneration as evidenced by increased proteoglycan distance (Fig. 6a, b**)** and cartilage thickness (Fig. 5a, b**).** Notably, ALN treatment before altered loading led to even lower levels of cartilage degeneration markers when compared to unloaded controls (*MMP13:* *p* < 0.001, Fig. 7a, b. *ADAMTS5:* *p* < 0.01, Fig. 8a, b). Our data suggest that pharmacologic inhibition of MMP13 and ADAMTS5 by ALN is an effective strategy to alleviate/slow down cartilage degeneration with altered loading of TMJ in OI mice model.

## Discussion

To the best of our knowledge, this is the first research studying the effects of mechanical loading on the osteochondral tissue of TMJ in osteogenesis imperfecta mouse model. Using multiple approaches, we examined the TMJ of both male and female *oim* mice under normal or altered loading, as well as the effects of ALN pretreatment on altered loading. Our key findings are as follows: (1) the altered loading of TMJ leads to sharp reduction in the bone volume fraction and tissue density of the subchondral bone, however, this catabolic effect can be prevented by the administration of ALN prior to loading; (2) the altered loading predisposes the mandibular condylar cartilage to early breakdown with decreased cartilage thickness and less proteoglycan distance, however, ALN treatment before altered loading can slow down this cartilage degeneration, possibly through an inhibitory effect on MMP13 and ADAMTS5; (3) the TMJ osteochondral changes in response to altered loading are comparable in male and female OI mice, meanwhile, the effects of alendronate pretreatment on altered loading are not gender-dependent.

### Effects of loading and alendronate on subchondral bone

Understanding how the TMJ osteochondral tissue (cartilage and subchondral bone) adapts to mechanical loads is a continuing challenge to scientists and the clinicians. Loading of TMJ in normal murine model has been shown to increase both the bone volume and the tissue density [19,22], as the adaption to compressive load via surface osteoblast and osteoclasts results in increased appositional growth. However, in contrast, *oim* mice exhibited a significantly decreased subchondral bone volume and density upon compressive loading. This might be associated with the compromised mechanosensing and mechanotransduction in OI skeleton, which has been proved in both patients and animal model [24]. First, osteocytes are the main bone cell type responsible for sensing mechanical strain, orchestrating signals of resorption and formation, and building the mineralized bone structures [25–27]. However, the osteocytes in *oim* mice are malformed with more spherical shaped osteocyte lacunae and increased lacunar density, which compromises the mechanical integrity and the mechanosensing and transduction [28]. Similar osteocyte defects were also reported in other OI mouse models [29]. Second, profound muscle weakness was identified in *oim* mice [24,30], and compromised muscle–bone unit impacts mechanosensing. By breeding heterozygote + /*oim* mice to heterozygote myostatin deficiency (+ /*mstn*) mice, the congenic double heterozygote (+ /*mstn* + /*oim*) mice displayed greater body weight, muscle mass, bone volume, and biomechanical strength than their + /*oim* littermates, indicating a likely role of myostatin deficiency in rescuing the defective muscle-bone unit and improving the mechanotransduction in OI condition. Together, it is possible that the comprised mechanosensing and mechanotransduction in *oim* mice due to defective bone-muscle unit abolishes the anabolic effect of compressive loading on TMJ. Of note, even if a mechanical stimulus promotes bone formation in OI mice, the collagen produced is still defective and weakened matrix remains uncorrected [31].

Alendronate (bisphosphonates) is a commonly prescribed to treat OI. It binds to calcium of the subchondral bone, and is released from the acidified bone surface and then taken up by multinucleated osteoclasts. ALN induces the apoptosis of osteoclasts and thus inhibit bone resorption [32–36]. ALN treatment has shown the ability to retain subchondral bone and prevent osteochondral tissue degeneration following the initiation of knee osteoarthritis in multiple animal models [37–40]. Interestingly, the efficiency of bisphosphonates treatment for alleviating matrix degeneration may depend on several systemic and environmental factors, such as the rate of subchondral bone turnover; specifically, ALN has been shown to be more effective with high bone turnover [41].In *oim* model, the collagen ultrastructure and cross linking is affected and bone turnover is increased with enhanced osteoclastic activity. Thus, the pretreatment of ALN has effectively prevented the subchondral bone loss in *oim* mice under altered loading **(**Figs. 2 and 3**)**. Furthermore, ALN directly interferes with osteoclastic phase of bone remodeling cycle (reducing bone resorption) [34–36] and indirectly stimulates bone formation [42] and that could be another plausible reason of increased bone volume fraction and mineralization.

Increased mineralization activity (alizarin intensity, Fig. 4) in ALN + Loaded group paralleled decreased osteoclastic activity and increased bone volume. ALN effects has been deeply studied on osteoclasts [43]; however, ALN is also known to have an indirect effect on bone formation by increasing the proliferation of osteoblasts residing on the surface [43,44]. In summary, ALN pretreatment substantially prevented the subchondral bone loss in OI male and female mice due to altered loading.

### Effects of loading and alendronate on mandibular condylar cartilage

Cartilage degeneration is characterized by degradation of extracellular matrix and is primarily due to imbalance between the synthesis and metabolism of the extracellular matrix [45]. Mechanical loading of the osteochondral tissue in compressive manner has been shown to be anabolic (increased cartilage thickness, chondrocyte proliferation and increased proteoglycan area) [19–22]. In this study of OI mice, altered loading with ALN pretreatment resulted in increased cartilage thickness and proteoglycan area (Figs. 5 and 6). This agrees with a previous in vitro study where ALN treatment increases the expression of extracellular matrix related genes (Col2a1, Col9a2 and Aggrecan) by increasing the Sox-9 expression, a central regulator for proliferation of chondrocytes and extracellular matrix genes [46].

MMP13 [47] and ADAMTS5 [48,49] are established cartilage degeneration markers [45] and upregulation of MMP13 [47] and ADAMTS5 [48,49] play an important role in cartilage degeneration [45,47,49–52]. In particular, MMP13 plays a central role in degrading collagenase matrix [53,54], whereas ADAMTS5 is known to degrade aggrecan [55,56]. Increased expression of MMP13 signifies the active cartilage diseases and precedes cartilage degeneration and ALN treatment has been shown to reduce the MMP13 expression in knee articular cartilage^45,47^. In our study, MMP13 and ADAMTS5 expressions in ALN + Loaded group were significantly lower than Saline + Loaded and unloaded control groups (Figs. 7 and 8), indicating that ALN may downregulate key cartilage degeneration markers, which possibly explained the restoration of cartilage thickness and proteoglycan distance by ALN (Figs. 5 and 6).Overall, these observations lead us to conclude that TMJ cartilage was very responsive to ALN treatment in OI TMJ loading model. Notably, ALN administration before the loading of the TMJ was able to prevent the degradation of the extracellular matrix and was anabolic to cartilage. We observed increased cartilage thickness and proteoglycan distance and decreased MMP13 and ADAMTS5.

Our study had several strengths including the analyzing both male and female OI mice, and in-depth histological and micro-CT examination. To the best of our knowledge, this is the first study looking at the effects of ALN on compressive loading of the osteochondral tissue in an OIM model. Alendronate and other bisphosphonates therapy have been used successfully to treat OI individuals and have significantly improved the quality of life in children. However, bisphosphonates may lead to decreased bone remodeling, decreased resorption of the growth plate cartilage in children’s and delayed healing of the osteotomy sites. Our future studies will focus on using older mice (6–8-month-old) using anti-TNFα as a therapeutic, targeting RANK/RANKL signaling pathway.

## Conclusion

Our present study demonstrated that ALN pretreatment can prevent the detrimental effects of loading on the osteochondral tissue of TMJ in OI mice model. Future research should unravel the signaling pathways involved in this process. Better understanding of the biological events during the loading of OI TMJ will help us identify therapeutic targets and provide tailored treatment options to the patients with OI.

## Funding Sources

Research reported in this publication was supported by the National Institute of Dental and Craniofacial Research of the National Institute of Health under Award Number KO8DE025914, RO3DE030526, RO3DE030226 to SY and by the American Association of Orthodontic Foundation provided to SY.

## Declarations

### Availability of data and material

The datasets underlying this article are available upon reasonable request to the corresponding author.
